# Supplementary material for: Attributable Mortality of Hip Fracture in Older Patients: A Retrospective Observational Study
Source: J Clin Med. 2020 Jul 24;9(8):2370. doi: 10.3390/jcm9082370 (PMC7465479; doi:10.3390/jcm9082370)
Supplement: Supplementary file 1 [file jcm-09-02370-s001.pdf]

Supplementary Materials

Text S1. STROBE Statement—Checklist of items that should be included in reports of *cohort studies.*

	Item No	Recommendation	Page No
**Title and abstract**	1	(*a*) Indicate the study's design with a commonly used term in the title or the abstract	Page 1
		(*b*) Provide in the abstract an informative and balanced summary of what was done and what was found	Page 1-2
		**Introduction**	
Background/rationale	2	Explain the scientific background and rationale for the investigation being reported	Page 3
Objectives	3	State specific objectives, including any prespecified hypotheses	Page 3
		**Methods**	
Study design	4	Present key elements of study design early in the paper	Page 4
Setting	5	Describe the setting, locations, and relevant dates, including periods of recruitment, exposure, follow-up, and data collection	Page 4
Participants	6	(*a*) Give the eligibility criteria, and the sources and methods of selection of participants. Describe methods of follow-up	Page 4
		(*b*) For matched studies, give matching criteria and number of exposed and unexposed	
Variables	7	Clearly define all outcomes, exposures, predictors, potential confounders, and effect modifiers. Give diagnostic criteria, if applicable	Page 4-6
Data sources/ measurement	8*	For each variable of interest, give sources of data and details of methods of assessment (measurement). Describe comparability of assessment methods if there is more than one group	Page 4-6
Bias	9	Describe any efforts to address potential sources of bias	Page 6-7
Study size	10	Explain how the study size was arrived at	Page 6
Quantitative variables	11	Explain how quantitative variables were handled in the analyses. If applicable, describe which groupings were chosen and why	Page 6-7
Statistical methods	12	(*a*) Describe all statistical methods, including those used to control for confounding	Page 6-7
		(*b*) Describe any methods used to examine subgroups and interactions	
		(*c*) Explain how missing data were addressed	
		(*d*) If applicable, explain how loss to follow-up was addressed	
		(*e*) Describe any sensitivity analyses	
		**Results**	
Participants	13*	(a) Report numbers of individuals at each stage of study—eg numbers potentially eligible, examined for eligibility, confirmed eligible, included in the study, completing follow-up, and analysed	Page 7 Figure 2
		(b) Give reasons for non-participation at each stage	
		(c) Consider use of a flow diagram	
Descriptive data	14*	(a) Give characteristics of study participants (eg demographic, clinical, social) and information on exposures and potential confounders	Page 7 Table 1
		(b) Indicate number of participants with missing data for each variable of interest	
		(c) Summarise follow-up time (eg, average and total amount)	

Outcome data	15*	Report numbers of outcome events or summary measures over time	Page 10 Table 2,3
Main results	16	(*a*) Give unadjusted estimates and, if applicable, confounder-adjusted estimates and their precision (eg, 95% confidence interval). Make clear which confounders were adjusted for and why they were included	Page 13 Table 4 and Figure 3
		(*b*) Report category boundaries when continuous variables were categorized	
		(*c*) If relevant, consider translating estimates of relative risk into absolute risk for a meaningful time period	
Other analyses	17	Report other analyses done—eg analyses of subgroups and interactions, and sensitivity analyses	Page 13 Figure 3 Supplement
**Discussion**			
Key results	18	Summarise key results with reference to study objectives	Page 16
Limitations	19	Discuss limitations of the study, taking into account sources of potential bias or imprecision. Discuss both direction and magnitude of any potential bias	Page 17
Interpretation	20	Give a cautious overall interpretation of results considering objectives, limitations, multiplicity of analyses, results from similar studies, and other relevant evidence	Page 16-17
Generalisability	21	Discuss the generalisability (external validity) of the study results	Page 16-17
**Other information**			
Funding	22	Give the source of funding and the role of the funders for the present study and, if applicable, for the original study on which the present article is based	Page 19

*Give information separately for exposed and unexposed groups.

Note: An Explanation and Elaboration article discusses each checklist item and gives methodological background and published examples of transparent reporting. The STROBE checklist is best used in conjunction with this article (freely available on the Web sites of PLoS Medicine at http://www.plosmedicine.org/, Annals of Internal Medicine at http://www.annals.org/, and Epidemiology at http://www.epidem.com/). Information on the STROBE Initiative is available at http://www.strobe-statement.org.

Text S2. Management in UPOG.

Management strategy in UPOG (previously detailed)[1] focused on early mobilization with the aim of chair-sitting and walking within 24 and 48 hours after arrival respectively, pain management using acetaminophen and morphine, the provision of air-filled mattresses for patients with pressure sores or a high risk of pressure sores as evaluated by the Braden scale (8), swallowing disorders detected using a systematic medical survey, detection of stool impaction and urinary retention using bedside ultrasound, correction of anemia with transfusion of packed red blood cells (usually when the hemoglobin level was <8 $g.L^{-1}$),[2] detection of delirium using the Confusion Assessment Method,[3] and malnutrition detection and management in conjunction with a nutritionist.

We recorded the preoperative hemoglobin level, and anemia was defined according to World Health Organization criteria.[4] We calculated preoperative serum creatinine and estimated creatinine clearance using the Cockcroft-Gault formula. Chronic renal failure was defined as a creatinine clearance ≤ 30 $ml.min^{-1}$.

Post-operative complications included postoperative delirium, need for physical restraints, stool impaction, urinary retention requiring drainage, pressure sore, infection, aspiration related to swallowing disorders, thromboembolic events, need for blood transfusion, cardiac insufficiency, myocardial infarction, acute atrial fibrillation, stroke, surgical complications, and admission into an intensive care unit (ICU).

References:

1. Boddaert, J.; Cohen-Bittan, J.; Khiami, F.; Le Manach, Y.; Raux, M.; Beinis, J.-Y.; Verny, M.; Riou, B. Postoperative admission to a dedicated geriatric unit decreases mortality in elderly patients with hip fracture. *PLoS ONE* **2014**, *9*, e83795, doi:10.1371/journal.pone.0083795.
2. Zerah, L.; Dourthe, L.; Cohen-Bittan, J.; Verny, M.; Raux, M.; Mézière, A.; Khiami, F.; Tourette, C.; Neri, C.; Le Manach, Y.; et al. Retrospective evaluation of a restrictive transfusion strategy in older adults with hip fracture. *J Am Geriatr Soc* **2018**, *66*, 1151–1157, doi:10.1111/jgs.15371.
3. Inouye, S.K.; van Dyck, C.H.; Alessi, C.A.; Balkin, S.; Siegal, A.P.; Horwitz, R.I. Clarifying confusion: the confusion assessment method. A new method for detection of delirium. *Ann. Intern. Med.* **1990**, *113*, 941–948, doi:10.7326/0003-4819-113-12-941.
4. McLean, E.; Cogswell, M.; Egli, I.; Wojdyla, D.; de Benoist, B. Worldwide prevalence of anaemia, WHO Vitamin and Mineral Nutrition Information System, 1993-2005. *Public Health Nutr* **2009**, *12*, 444–454, doi:10.1017/S1368980008002401.

Text S3. Original statistical analysis plan.

VERSION	AUTHOR	SUMMARY OF CHANGES	DATE
**1**	David Hajage	First draft	2019-02-04
**2**	David Hajage	Revision 1	2019-04-09

1 Patients

From June 2009 to July 2018 (*i.e.* 108 months), all consecutive patients with hip fracture admitted to the UPOG were evaluated for eligibility. Patients were included if their primary presentation was due to hip fracture and if they were ≥70 years of age. Patients were excluded only if follow-up could not be obtained.

2 Primary endpoint (response variable)

The primary endpoint is 6-month mortality status.

3 Descriptive variables

- Delay to first sitting (days)
- Delay to first walking (days)
- Length of stay in acute care (days)
- Immediate authorization for walking (yes/no)
- Length of stay in rehabilitation departments (days)
- Length of stay in acute care + rehabilitation (days)
- Outcome after UPOG (death, return home, rehabilitation center)
- Outcome after rehabilitation (death, return home)
- Outcome after 6 month (dead/alive).

4 Explanatory variables

Explanatory variables were classified a priori according to four domains:

a. Baseline characteristics

- Age, age ≥ 90 years
- Sex
- Home or nursing home (living in institution) condition
- All medications received before admission which are summarized by the number of medications taken.
- Previous medical history of:
    - obesity
    - dementia
    - diabetes
    - hypertension
    - cardiac failure
    - coronary artery disease
    - heart valve disease
    - atrial fibrillation
    - peripheral vascular disease
    - stroke
    - hemiplegia/paraglegia
    - cancer

- COPD
- alcohol abuse
- drug abuse
- blindness
- renal insufficiency (preoperative serum creatinine is measured and creatinine clearance is estimated using the Cockcroft formulae) defined by Cockcroft <30 ml/min
- denutrition, albumin
- Preoperative anemia defined following World Health Organization guidelines, hemoglobin value;

- Co-morbidity severity assessed using the Cumulative Illness Rating Scale (CIRS 52 score)
- Frailty assessed using the Rockwood score
- Functional status assessed using
  - the Activities of Daily Living (ADL) scale
  - and the Instrumental Activities of Daily Living (IADL) scale.
- Walking ability before the hip fracture (no walking disability, walking disability, does not walk)
- Type of hip fracture (Femoral neck or intertrochanteric fracture)

b. **Acute co-existing illness including acute conditions present before surgery that could have promoted the fall or be caused by the fall** (other traumatic lesion, acute coronary syndrome, stroke, infection). Patients are classified using two experts, when disagreement occurs a third expert is used. The kappa score is calculated.

c. Perioperative factors

- Duration of surgery
- Delay to surgery (≤24h, >48h)
- Type of surgery (gamma nail, dynamic hip screw, unipolar prosthesis, or bipolar prosthesis)
- Type of anesthesia (general (AG), or regional (ALR))
- Blood transfusion and number of packed red blood cells transfusion

d. Postoperative course

- Postoperative complications
  - Delirium/delirium requiring physical restraint
  - Swallowing disorders with aspiration
  - Stool impaction
  - Urinary retention requiring drainage
  - Pressure ulcer
  - Acute heart failure
  - Acute coronary syndrome
  - Paroxystic atrial fibrillation
  - Hemorrhage
  - Stroke
  - Infection
  - Venous thromboembolism
  - Surgical complication (redo surgery or infection)
  - Admission to intensive care unit

Postoperative complications are summarized by the Dindo-Clavien classification (from 0 to 5, 5 being complications resulting in death): presence of severe complication defined by Dindo-Clavien score from 3 to 5 (3: requiring surgical, endoscopic or radiological intervention; 4: life-threatening complications requiring intermediate care or intensive care unit; 5: death of patients).

Patients are classified using two experts, when disagreement occurs a third expert is used. The kappa score is calculated.

5 Statistical methods

Patient characteristics will be described overall and according to 6-month mortality status. Quantitative variables will be described by their mean, standard deviation, median, interquartile range, minimum, maximum value and number of missing data. Qualitative variables will be described by frequency, percentage and number of missing data. This descriptive analysis will be performed in the overall sample and in two subgroups: alive and dead at 6 months.
The model of prediction of 6-months mortality will be constructed in the sample with no missing values for all candidate explanatory variables.
In the spirit of parsimony and to keep the final model as simple as possible, we will

1) separately select the most important variables in each of the four domains,
2) and then fit the final model with all the variables selected in the previous step.

a. Separately select the most important variables in each domain

Separately for each domain, all continuous variables will be categorized, either through clinically relevant thresholds from the literature, or using ROC curve to determine the best threshold (maximization of the Youden index). Univariate comparison between survivors and dead patients will be performed, using Student t test, Mann-Whitney test, Chi square test, or Fisher's exact test, as appropriate. All variables will be included in a multivariate logistic model with 6-month mortality as the explained variable. OR and their corresponding 95% confidence interval (95%CI) will be provided for each variable. Discrimination and calibration of multivariable the model will be assessed using c-statistics and Hosmer-Lemeshow test. This model will be used to determine the most important variables of the corresponding domain.

b. Fit the final model

A final model will be constructed using the most important variables of each domain selected in the previous step. No further selection of variables will be performed. Again, discrimination and calibration will be evaluated, and OR will be provided with their 95%CI. Moreover, Averaged Attributable Fractions (AAF) will be computed for each variable and each domain.[1–3]

c. Sensitivity analyses

The final model and the AAF associated with each domain depend on the way the most important variables of each domain are selected in the first step. Several methods will be used:

- Selection of the significant variables of each domain-specific multivariate logistic model. Two thresholds will be used: $p < 0.05$ (primary analysis), and $p < 0.1$.
- Selection of the 3 variables with the greater AAF in each domain-specific multivariate logistic model.

The first method of selection ($p < 0.05$) and the corresponding final model and AAFs will constitute our primary analysis. The other methods will constitute the sensitivity analyses and will be reported as supplementary analysis.

All statistical analysis will be performed using R (version 3.5.1 or higher) software.

Refernces:

1. Eide, G.E.; Gefeller, O. Sequential and average attributable fractions as aids in the selection of preventive strategies. *J Clin Epidemiol* **1995**, *48*, 645–655, doi:10.1016/0895-4356(94)00161-i.

2. Ferguson, J.; Alvarez-Iglesias, A.; Newell, J.; Hinde, J.; O'Donnell, M. Estimating average attributable fractions with confidence intervals for cohort and case-control studies. *Stat Methods Med Res* **2018**, *27*, 1141–1152, doi:10.1177/0962280216655374.
3. Rückinger, S.; von Kries, R.; Toschke, A.M. An illustration of and programs estimating attributable fractions in large scale surveys considering multiple risk factors. *BMC Med Res Methodol* **2009**, *9*, 7, doi:10.1186/1471-2288-9-7.

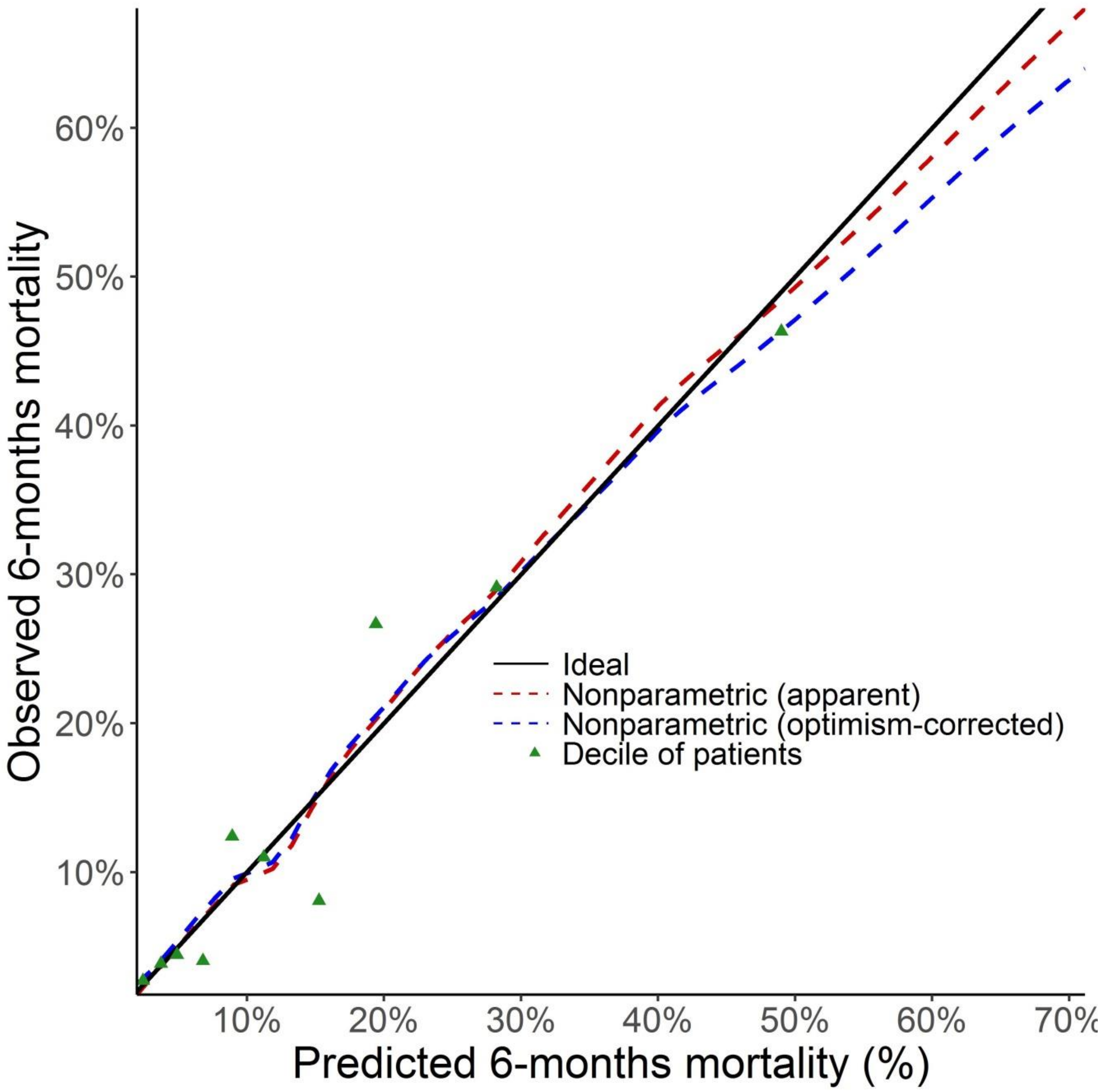

60%
50%
40%
30%
20%
10%
Observed 6-months mortality
10%
20%
30%
40%
50%
60%
70%
Predicted 6-months mortality (%)
Ideal
Nonparametric (apparent)
Nonparametric (optimism-corrected)
Decile of patients

(**A**)

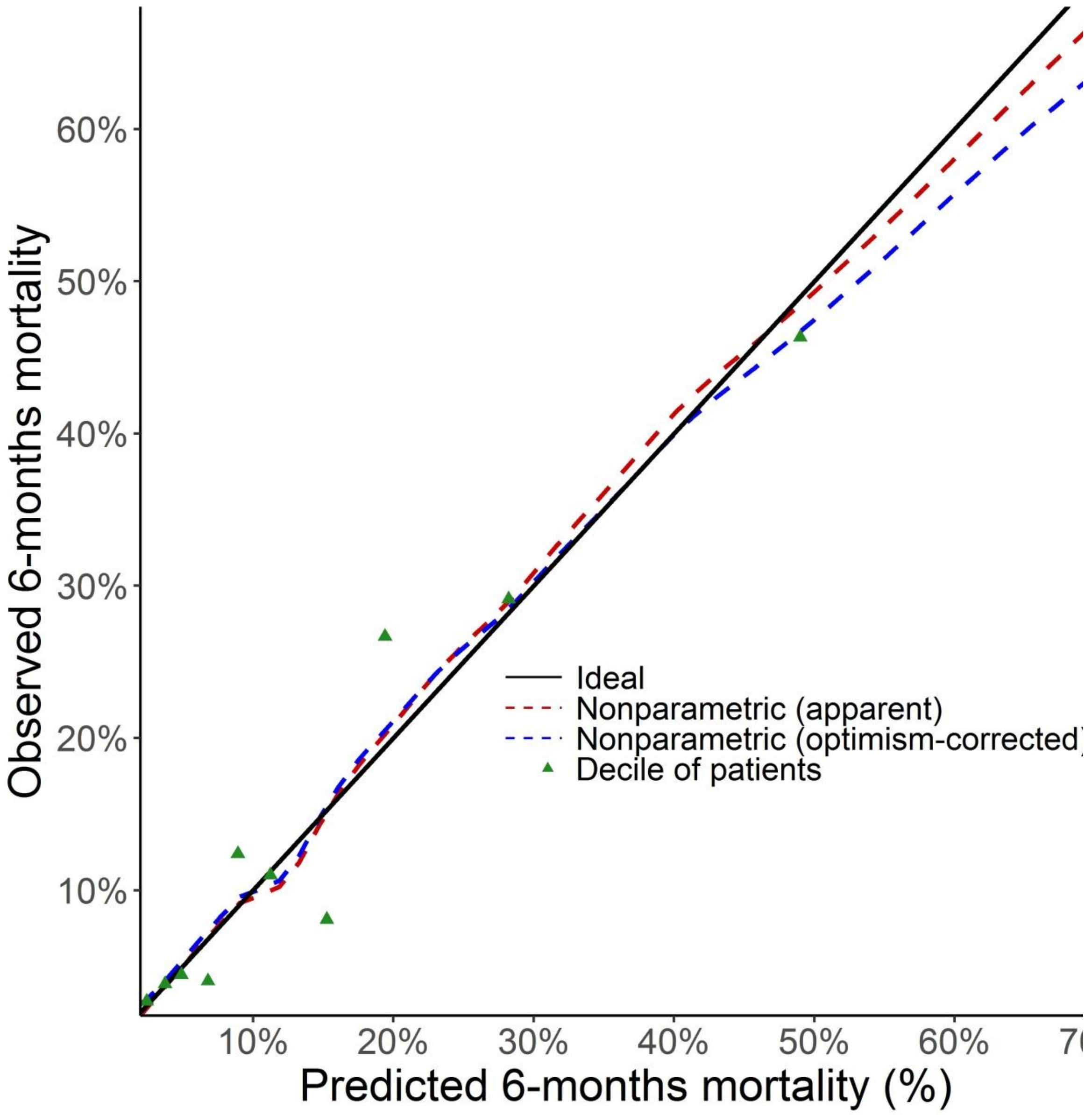
60%
50%
40%
30%
20%
10%
Observed 6-months mortality
10%
20%
30%
40%
50%
60%
Predicted 6-months mortality (%)
Ideal
Nonparametric (apparent)
Nonparametric (optimism-corrected)
Decile of patients

(**B**)

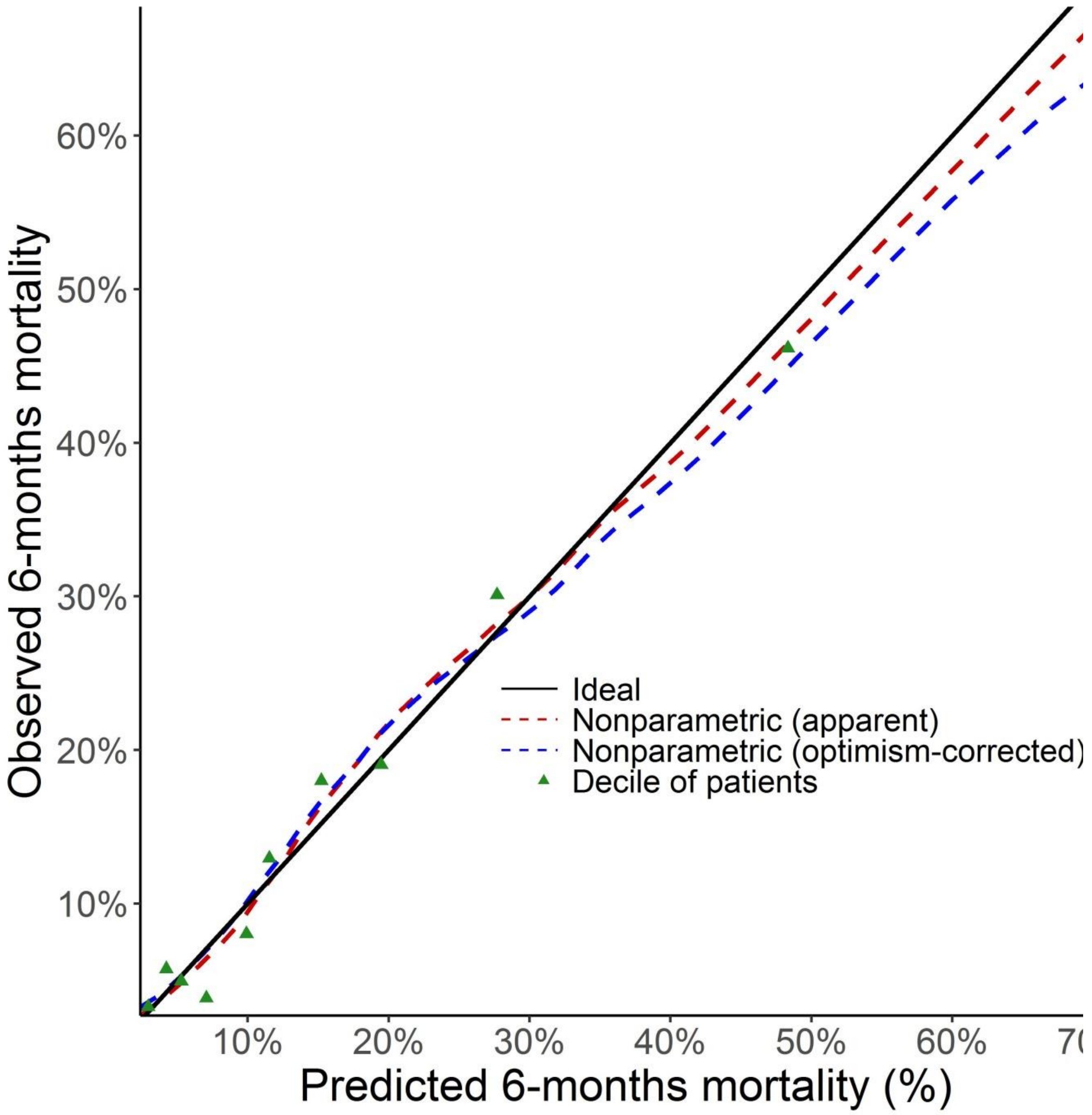

(**C**)

Figure S1. Apparent and optimism-corrected calibration plots for the internal validation of the 3 models. (**A**): selection of the variables with $p < 0.05$ of each domain-specific multivariate logistic model. (**B**): selection of the variables with $p < 0.1$ of each domain-specific multivariate logistic model. (**C**): selection of the 3 variables with the greater AAF in each domain-specific multivariate logistic model. AAF = Averaged Attributable Fractions

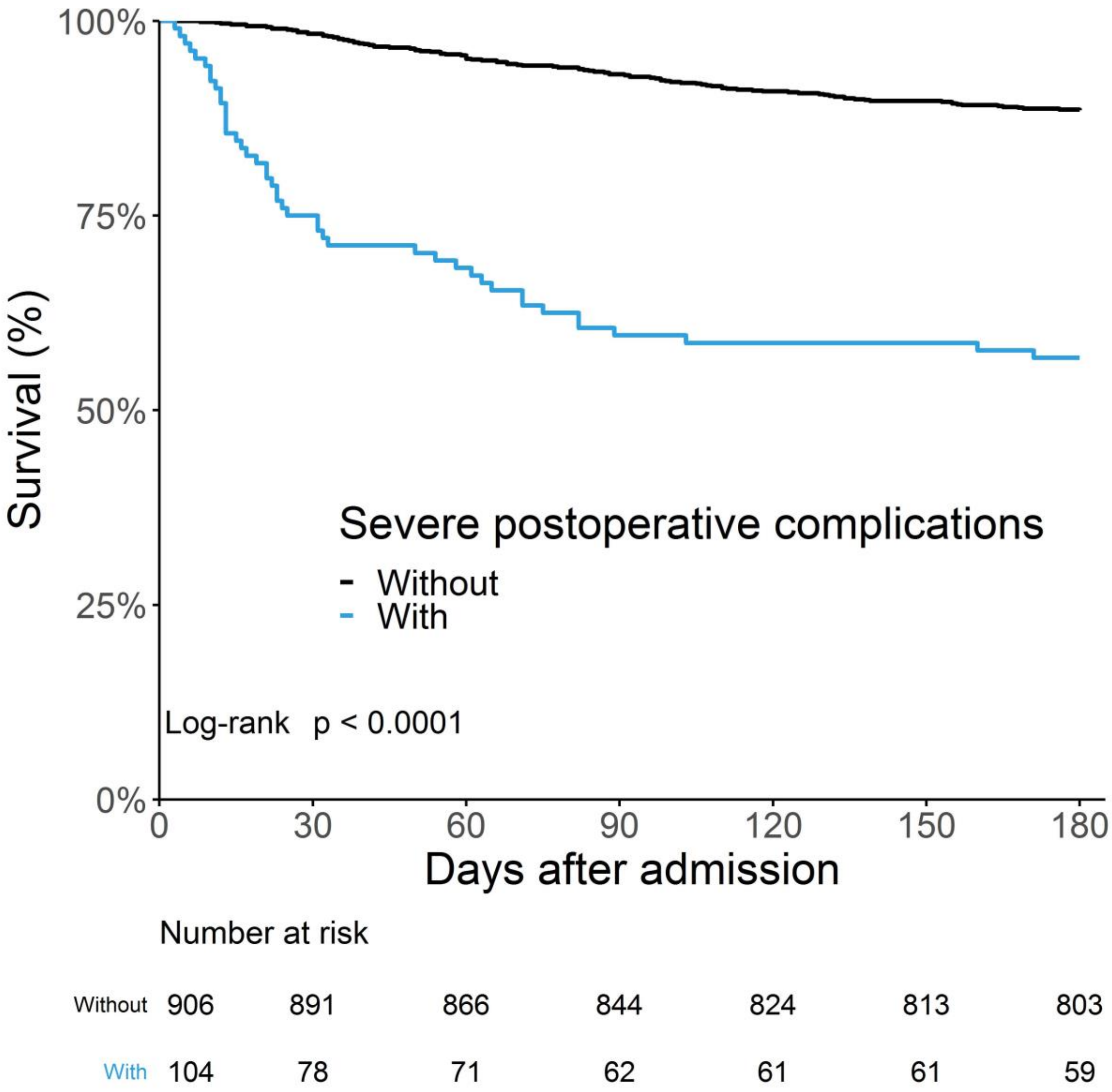
100%
75%
50%
25%
0%
Survival (%)
Severe postoperative complications
Without
With
Log-rank p < 0.0001
0
30
60
90
120
150
180
Days after admission
Number at risk
Without 906 891 866 844 824 813 803
With 104 78 71 62 61 61 59

(**A**)

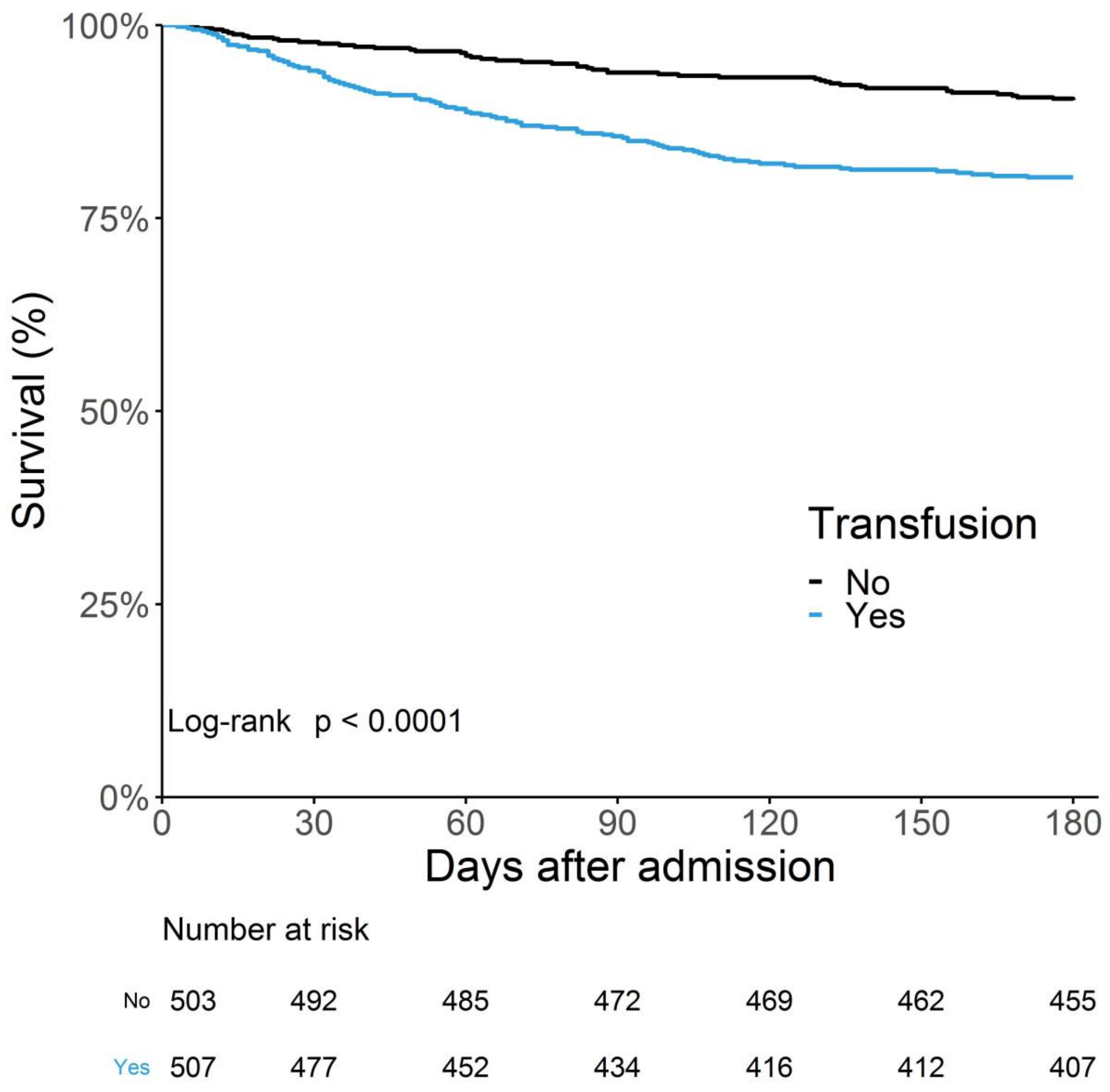
100%
75%
50%
25%
0%
Survival (%)
Transfusion
No
Yes
Log-rank p < 0.0001
0
30
60
90
120
150
180
Days after admission
Number at risk
No 503 492 485 472 469 462 455
Yes 507 477 452 434 416 412 407

(**B**)

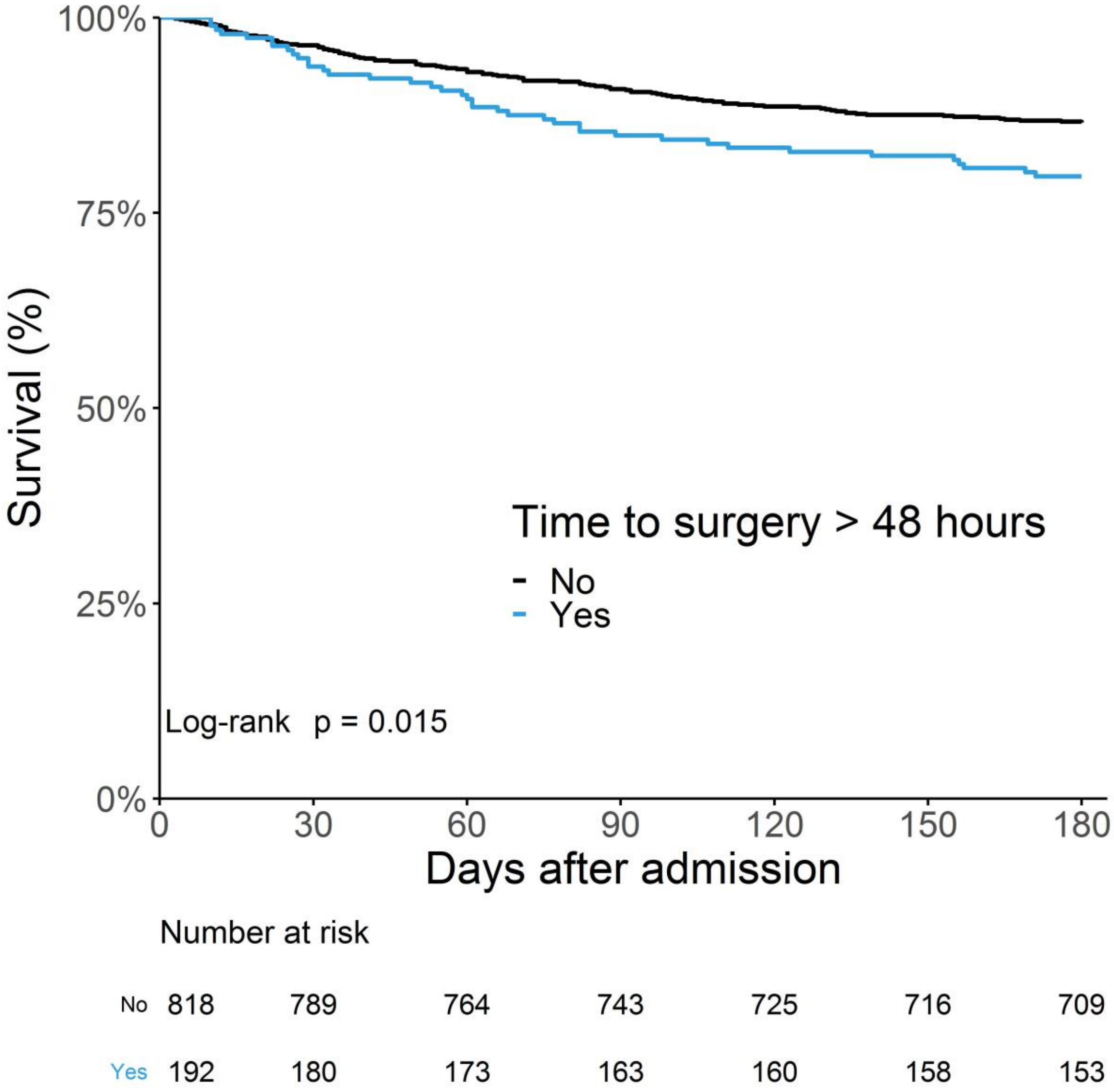

(**C**)

Figure S2. Non-adjusted survival curves for death in patients. (**A**) without (n=906) or with severe postoperative complications (Dindo-Clavien score ≥ 3, n=104); *P* value refers to between-group comparison with the log-rank test. (**B**) without (n = 503) or with (n = 507) perioperative transfusion. *P* value refers to between-group comparison with the log-rank test. (**C**) with time to surgery > 48 hr (n = 192) or ≤ 48 hr (n = 818). *P* value refers to between-group comparison with the log-rank test.

Table S1. The intermediate multivariate logistic model predicting 6-month mortality with baseline characteristics.

Variables	OR [95% CI]	P value	AAF (%) [95% CI]
**Baseline characteristics**			
Age, ref < 88 years			
• Age ≥ 88 years	1.80 [1.20 – 2.60]	.002	13.9 [5.0 – 22.8]
Gender, ref = F			
• Gender = M	2.01 [1.33 – 3.04]	.011	9.2 [2.9 – 15.5]
CIRS, ref < 11			
• CIRS ≥ 11	2.49 [1.72 – 3.61]	< .0001	20.5 [10.8 – 30.3]
ADL, ref ≥ 5.5			
• ADL < 5.5	2.51 [1.71 – 3.68]	< .0001	23.9 [13.5 – 34.4]
Femoral neck fracture, ref = No			
• Femoral neck fracture = Yes	0.67 [0.46 – 0.97]	.035	10.6 [0.6 – 21.8]
		**Total**	**78.3 [63.4 – 93.1]**

Baseline characteristics: N = 1010, C-Index = 0.723 95% CI [0.679 to 0.767]. Abbreviations: AAF = Averaged attributable factor; ADL: Activities of daily living scale; CIRS: cumulative illness rating scale; OR = Odds ratio; CI: confidence interval; ref = reference value; F = female, M: Male.

Table S2. The intermediate multivariate logistic model predicting 6-month mortality with acute co-existing illness.

Variables	OR [95% CI]	P value	AAF (%) [95% CI]
**Acute co-existing illness**			
Acute co-existing illness, ref = no			
• Acute co-existing illness = yes	1.04 [0.59 – 1.83]	.89	4 [- 15 – 16]
		**Total**	**4 [- 15 – 16]**

Acute co-existing illness: N = 1010, C-index = 0.502 CI95%[0.475 to 0.529]. Abbreviations: AAF = Averaged attributable factor; OR = Odds ratio; CI: confidence interval.

Table S3. The intermediate multivariate logistic model predicting 6-month mortality with perioperative factors.

Variables	OR [95% CI]	P value	AAF (%) [95% CI]
**Perioperative factors**			
Time to surgery, ref ≤ 48 hours			
• Time to surgery > 48 hours	1.67 [1.11 – 2.52]	.014	7.7 [1.5 – 14.4]
Transfusion, ref = No			
• Transfusion = yes	2.3 [1.59 – 3.32]	< .0001	32.9 [18.5 – 47.3]
		**Total**	**40.6 [25.7 – 55.5]**

Perioperative factors: N = 1010, C-Index = 0.621 95% CI [0.575 to 0.666]. Abbreviations: AAF = Averaged attributable factor; OR = Odds ratio; CI: confidence interval.

Table S4. Complications quoted as severe (Dindo-Clavien score 3 to 5) and observed during the acute stay (n=104).

Complications	Number of patients (%)
Infectious pneumonia	33 (32)
Acute coronary syndrome	31 (30)
STEMI	8
Non STEMI	23
Acute pulmonary edema (cardiac insufficiency)	30 (29)
Gastrointestinal hemorrhage	18 (17)
Other severe infections	11 (11)
Redo surgery	9 (9)
Pulmonary embolism	7 (7)
Acute renal failure	7 (6)
Cardiac rhythm disturbances	5 (5)
Other hemorrhage/hematoma	3 (3)
Cardio circulatory arrest	3 (3)
Urinary obstacle	3 (3)
Occlusion	2 (2)
Other complications	13 (12)

Because a given patient may have several severe complications, the sum of percentages do not add to 100%. Abbreviations: STEMI: ST segment elevation myocardial infarction.

Table S5: The intermediate multivariate logistic model predicting 6-month mortality with postoperative complications

Variables	OR [95% CI]	P value	AAF (%) [95% CI]
**Postoperative complications**			
Dindo-Clavien score, ref < 3			
• Dindo-clavien score ≥ 3	5.88 [3.78 – 9.12]	< .001	22.2 7.9 – 36.4]
		**Total**	**22.2 7.9 – 36.4]**

Acute co-existing illness: N = 1010, C-index = 0.502 CI95%[0.475 to 0.529]. Abbreviations: AAF = Averaged attributable factor; OR = Odds ratio; CI: confidence interval.

Table S6. Estimates of the 6-month attributable mortality according to each domain (sensitivity analyses: selection of the variables with P<0.10 for each domain-specific multivariate logistic model).

Variables	OR [95% CI]	*P* value	AAF (%) [95% CI]
**Baseline characteristics → AAF = 62.4**			
Age, ref < 88 years			
• Age ≥ 88 years	1.72 [1.16 – 2.56]	.007	10.8 [2.7 – 19.0]
Gender, ref = Female			
• Gender = Male	1.93 [1.25 – 2.97]	.003	7.3 [1.8 – 12.7]
CIRS, ref < 11			
• CIRS ≥ 11	2.27 [1.55 – 3.35]	< .001	15.6 [7.0 – 24.2]
ADL, ref ≥ 5.5			
• ADL < 5.5	2.52 [1.68 – 3.76]	< .001	20.0 [1.0 – 30.1]
Femoral neck fracture, ref = No			
• Femoral neck fracture = Yes	0.67 [0.45 – 1.01]	.054	8.7 [1.7– 19.0]
**Co-existing acute illness → AAF = 0**			
**Perioperative factors → AAF = 12.3**			
Time to surgery, ref ≤ 48 hours			
• Time to surgery > 48 hours	1.36 [0.87 – 2.13]	.18	2.7 [1.0 – 6.5]
Transfusion, ref = No			
• Transfusion = yes	1.53 [1.02 – 2.28]	.04	9.6 [1.1 – 20.4]
**Postoperative factors → AAF = 11.9**			
Dindo-Clavien score, ref < 3			
• Dindo-clavien score ≥ 3	4.91 [3.06 – 7.90]	< .001	11.9 [7.1 – 16.8]
		**Total**	**86.6 [75.3 – 98.0]**

N = 1010; C-index = 0.78 95% CI [0.74 to 0.82]; Hosmer-Lemeshow test: *X2* = 10.737, ddl=8, *P* = .22. Co-existing acute illness factors were not included in the final model because it was not significant in univariate analysis. Abbreviations: AAF = Averaged attributable fraction, ADL: Activities of daily living scale, CIRS: cumulative illness rating scale; OR = Odds ratio; CI: confidence interval; ref = reference value.

Table S7. Estimates of the 6-month attributable mortality according to each domain (sensitivity analyses: selection of the 3 variables with the greater AAF in each domain-specific multivariate logistic model.).

Variables	OR [95% CI]	*P* value	AAF (%) [95% CI]
**Baseline characteristics → AAF = 47.7**			
Age, ref < 88 years			
• Age ≥ 88 years	1.55 [1.06 – 2.28]	.024	9.4 [1.1 – 17.6]
CIRS, ref < 11			
• CIRS ≥ 11	2.54 [1.73 – 3.71]	< .0001	18.6 [8.7 – 28.6]
ADL, ref ≥ 5.5			
• ADL < 5.5	2.33 [1.57 – 3.47]	< .0001	19.7 [9.5 – 29.9]
**Co-existing acute illness → AAF = 8.4**			
Acute factor, ref = No			
• Acute factor= yes	0.79 [0.42 – 1.5]	.477	8.4 [11.3 – 28.2]
**Perioperative factors → AAF = 14.9**			
Time to surgery, ref ≤ 48 hours			
• Time to surgery > 48 hours	1.35 [0.86 – 2.1]	.187	28.8 [2.3 – 7.9]
Transfusion, ref = No			
• Transfusion = yes	1.65 [1.11 – 2.45]	.012	12.1 [1.6 – 22.6]
**Postoperative factors → AAF = 12.8**			
Dindo-Clavien score, ref < 3			
• Dindo-clavien score ≥ 3	4.95 [3.09 – 7.92]	< .0001	12.6 [7.3 – 17.9]
First step authorized by the surgeon at the end of surgery (Day 1), ref = yes			
• First step = no	1.08 [0.45 – 2.57]	.862	0.2 [- 2.1 – 2.4]
		**Total**	**83.8 [68.9 – 98.7]**

N = 1010; C-index = 0.77 95% CI [0.72 to 0.81]; Hosmer-Lemeshow test: $X2$ = 3.905, ddl=8, P = .87. Co-existing acute illness factors were not included in the final model because it was not significant in univariate analysis. Abbreviations: AAF = Averaged attributable fraction, ADL: Activities of daily living scale, CIRS: cumulative illness rating scale; OR = Odds ratio, CI: confidence interval; ref = reference value.
